# PAlarm: a pediatric clinical worsening identification system

**DOI:** 10.1186/1824-7288-40-S1-A51

**Published:** 2014-08-11

**Authors:** Viviana Frigato, Liliana Vagliano, Maria Cristina Rossi

**Affiliations:** 1 Città della Salute e della Scienza Hospital of Turin - Regina Margherita Children Department, Italy; 2 University of Turin – Pediatric Nursing Degree Course Italy; 3 Città della Salute e della Scienza Hospital of Turin – Management of In Hospital Emergency Working Group, Italy

## 

Rapid Response System (RRS) is by now commonly used and internationally validate to manage in hospital emergency. Both its optimal management and early identification are essential to reduce mortality and neurological sequences of anoxia.

However, in the pediatric Italian population, this system is poorly employ, even if its effectiveness has already been demonstrated.

Therefore, in 2010 at Turin a study [1] was conducted in 5 units of a third level pediatric hospital; the aim was to experiment the only pediatric system completely validate in literature (the Pediatric early Warning Score System). In order to apply this system, it was necessary to adapt it at local reality, analyzing relevant cases (PEWS≥ 3); all nursing staff underwent an interview to analyze utility and effectiveness of the system.

The obtained results confirm the hypothesis that a PEWS system is suitable for general departments where respiratory pathologies (main cause of death in pediatric age) prevail.

Thanks to this pilot study, the Regina Margherita Children Hospital of Turin has developed the application of this system at the whole hospital, further adjusting it in order to improve the adaptation at local reality, based on preliminary results. The new system has been called PAlarm (Pediatric Alarm), to better connect with METal methodology broadly used for adult patient all over Italian country.

Moreover, specific educational courses has been developed, both for nurses and physicians, in order to correctly apply this system in journal clinical activity.
